# Editorial: The Role of DNA Repair Pathways in Resistance to Chemotherapy and Radiotherapy in Cancer

**DOI:** 10.3389/fonc.2022.894357

**Published:** 2022-04-08

**Authors:** David Y. Lee, Rosa María Bermúdez-Cruz, José Díaz-Chávez

**Affiliations:** ^1^Department of Internal Medicine, Division of Hematology/Oncology, Section of Radiation Oncology, University of New Mexico School of Medicine and Comprehensive Cancer Center, Albuquerque, NM, United States; ^2^Departamento de Genética y Biología Molecular, Centro de Investigación y de Estudios Avanzados (CINVESTAV-IPN), Ciudad de México, Mexico; ^3^Unidad de Investigación Biomédica en Cáncer, Instituto de Investigaciones Biomédicas, Universidad Nacional Autónoma de México (UNAM)/Instituto Nacional de Cancerología (INCan), Ciudad de México, Mexico

**Keywords:** DNA repair pathways, cancer, chemoresistance, radioresistance, DNA damage response

## Introduction

Over the past decade, there has been enormous progress in treating cancer patients with the continued development of novel targeted therapies (1), the advent of immunotherapy (2), and novel radiation therapy technologies (3). However, resistance to radiation therapy and chemotherapy continues to be a major problem in our field, for which many patients ultimately succumb to the disease. Although we celebrate the approval of each new targeted therapy, we invariable find that cancer cells develop resistance to each one. Furthermore, it is still very difficult to predict who will respond to immunotherapy. Therefore, the search for radiation therapy sensitizers continues.

Through our Research Topic, several principles have emerged that may guide us in the future to overcome this resistance. First, some biomarkers may predict who will be more resistant to radiation and/or chemotherapy. Zhang et al. showed that nasopharyngeal carcinoma patients with elevated levels of LCN2 (Lipocalin 2) showed resistance to radiation therapy. We could consider treatment intensification for these patients with LCN2 elevation by considering higher radiation doses or the addition of novel targeted therapies. Similarly, Fang et al. demonstrated that increased levels of NTGN1 (neuroligin 1) predicted resistance to cisplatin treatment in epithelial ovarian cancer cells, identifying a subgroup of patients for treatment intensification with additional systemic agents. Huang et al. utilized epithelial-mesenchymal transformation and DNA repair gene panels to classify colorectal cancer patients, which may guide treatment selection of chemotherapy vs immunotherapy to optimize treatment response.

Second, some pathways can ameliorate existing treatments by synergistic effect or through synthetic lethal interactions. Rose et al. prepared a wonderful review on the role of PARP inhibitors, specifically in the setting of tumors harboring BRCA1/2 mutations. The synthetic lethal interaction between PARP inhibitor and BRCA1/2 mutations represent one of the successful translation of basic research (4). Cancers with defects or mutations in the homologous recombination (HR) DNA repair pathway also respond to radiation therapy. Therefore, the PARP inhibitor and radiation therapy combination should be considered to obtain a durable response. Interestingly, Sabbatino et al. observed that patients with intrahepatic cholangiocarcinoma harboring BAP1 (BRCA1 Associated Protein 1) mutation may be sensitive to a PARP inhibitor. This is because BAP1 interacts with BRCA1, and BAP1 mutation likely alters the HR DNA repair pathway. Thus, consideration should be given for a potential role to PARP inhibitors in situations with alternations in the HR pathway, not just BRCA1/2 mutations.

The synergistic effect between temozolomide and mifepristone was shown by Llaguno-Munive et al. Mifepristone, an antihormonal agent, can enhance the effects of temozolomide by decreasing the levels of VEGF (vascular endothelial growth factor) and P-glycoprotein in murine orthotopic glioblastoma model. Since mifepristone would be repurposed for glioblastoma treatment, this drug represents a potential target for rapid clinical translation. Whether mifepristone and temozolomide can be combined with radiation therapy safely would be an important question to address. Similarly, Hong et al. demonstrated that the inhibition of thioredoxin reductase 1 by isodeoxyelephantopin synergistically enhanced the effect of cisplatin in colon cancer cells. Thus, the addition of new agents such as mifepristone or thioredoxin reductase 1 inhibitor to existing treatment can lead to synergistic effects and overcome or delay potential chemo/radiation resistance.

Third, there are potential novel pathways and inhibitors that can modulate the effect of radiation or chemotherapy. The role of non-coding RNAs and exosomes in radiation and chemotherapy response was addressed by Zhang et al. and Zhong et al., respectively. While a role for non-coding RNAs was shown in neck and head cancer radiotherapy, exosomes, vesicles which also transport non-coding RNAs plus protein are suggested to play a role in drug resistance in cancer. Concerning chemo/radiation resistance, these two areas of research, poorly studied, hold the potential to dramatically alter our understanding of chemo/radiation resistance. How the RAS oncogenic pathway impinges on the DNA repair pathway and subsequent therapeutic resistance is addressed by Caceres-Gutierrez et al. with the recent approval of RAS G12C mutant inhibitor (5), one could consider how this inhibitor could be combined with radiation therapy in lung and pancreas patients who frequently harbor this mutation. Wang et al. provide evidence for alternations in DNA damage repair pathways in esophageal squamous cell carcinomas, and Carlos-Reyes et al. describe biological adaptations of tumor cells to radiation therapy along with implications of such adaptation in patients outcome.

Avery et al. reviewed the role of GLI1 (glioma Family Zinc Finger 1) as a therapeutic target in cancer. One of the GLI1 inhibitors, Vismodegib, has shown a dramatic effect on unresectable basal cell carcinomas of the skin (6). Clinically, we are now able to convert some of the unresectable basal cell carcinoma patients to surgery by shrinking the tumor. Questions still remain on the duration of Vismodegib treatment before resistance develops, the extent of surgical resection, and radiation therapy’s role in the optimal management of basal cell carcinoma patients. Then, Lagunas-Rangel et al. provide a list of natural compounds that target DNA repair pathways. Currently, there has been significant difficulty in developing novel radiation therapy sensitizers, and the list of natural compounds provides an excellent starting point.

A fourth approach to sensitize drug-resistant cancers has been by including PDT (photodynamic therapy) in different types of cancer treatment (7). Gemcitabine has been described to cause DNA damage and is used to control hepatic cancer cells (8). Yang et al. in this particular case, have shown that cholangiocarcinoma cells resistant to gemcitabine and exposed to PDT display apoptosis, viability is reduced, and they are arrested in the G1 cell cycle phase.

In summary, our Research Topic has illuminated our understanding of radiation and chemotherapy resistance mechanisms, also some novel biomarkers to predict such resistance, novel pathways that interact by synergistic or synthetic lethal interactions, and potential inhibitors and pathways that may enhance the effect of radiation and/or chemotherapy.

## Author Contributions

All authors listed have made a substantial, direct, and intellectual contribution to the Research Topic and approved it for publication.

## Conflict of Interest

The authors declare that the research was conducted in the absence of any commercial or financial relationships that could be construed as a potential conflict of interest.

## Publisher’s Note

All claims expressed in this article are solely those of the authors and do not necessarily represent those of their affiliated organizations, or those of the publisher, the editors and the reviewers. Any product that may be evaluated in this article, or claim that may be made by its manufacturer, is not guaranteed or endorsed by the publisher.
